# Carcinosarcoma of the parotid gland: a case report and review of the literature

**DOI:** 10.1186/s13256-023-04280-7

**Published:** 2024-01-20

**Authors:** Swachi Jain, Mohammed Abdelwahed, Daniel Hector Chavarria, Lucio Pereira, Gary Stone, Alan Johnson, Jian Yi Li

**Affiliations:** 1 Department of Pathology and Laboratory Medicine, North Shore University Hospital and Long Island Jewish Medical Center, Donald and Barbara Zucker School of Medicine at Hofstra/Northwell Health, 2200 Northern Blvd, Suite 104, Greenvale, NY 11548 USA; 2 Department of Otolaryngology, Head and Neck Surgery, Long Island Jewish Medical Center, Donald and Barbara Zucker School of Medicine at Hofstra/Northwell Health, New Hyde Park, NY USA; 3 Department of Pathology, Huntington Hospital, Northwell Health, Huntington, NY USA; 4 Department of Diagnostic Radiology, Long Island Jewish Medical Center, Donald and Barbara Zucker School of Medicine at Hofstra/Northwell Health, New Hyde Park, NY USA

**Keywords:** Parotid gland, Salivary gland neoplasm, Carcinoma, Sarcoma, Carcinosarcoma, Milan classification, Liposarcoma

## Abstract

**Background:**

Carcinosarcoma of the parotid gland is an extremely rare malignancy comprising of 0.04–0.16% of all salivary gland tumors. This is the first case of an adenoid cystic carcinoma with chondrosarcoma to the best of our knowledge. They consist of distinct carcinomatous and sarcomatous components and may arise de novo or from a preexisting pleomorphic adenoma.

**Case presentation:**

Herein we present a case of an 80-year-old white female who presented with progressively increasing left facial swelling over 6 weeks. Magnetic Resonance Imagining revealed a mass (3.4 cm) in the parotid gland with a predominant cystic/necrotic component. The cytology was atypical (Milan3) and a total parotidectomy and selective lymph node dissection was done. The resection showed extensive necrosis with high grade sarcomatous (chondrosarcoma) areas. The epithelial component was adenoid cystic carcinoma with perineural invasion. The patient is currently undergoing radiotherapy of the tumor bed and skull base due to propensity of perineural invasion of the adenoid cystic component. The most common carcinomas in carcinosarcomas of salivary glands are adenocarcinoma and squamous cell carcinoma.

**Conclusion:**

Carcinosarcoma is a high-grade aggressive lesion with a poor prognosis and should be treated aggressively. More studies are needed to understand the origin of these tumors.

## Introduction

Carcinosarcoma or malignant mixed tumor of the salivary gland is a biphasic tumor having distinct carcinomatous and sarcomatous components [1]. They are extremely rare and consist of 0.04%–0.16% of all salivary gland tumors [2]. These tumors most commonly are seen in the parotid gland and may arise de novo or from a pre-existing pleomorphic adenoma. The most common carcinomas are adenocarcinoma, undifferentiated carcinoma, and squamous cell carcinoma [3]. We herein present a case with adenoid cystic carcinoma being the carcinomatous component with chondrosarcoma and is the first such case to the best of our knowledge.

## Case presentation

### Patient information

An 80-year-old white female presented to the hospital with left lower facial swelling progressively increasing in size over the past two months. There was no history of dysphagia, odynophagia, fever, or weight loss. There was no history of lesions in the parotid gland.

### Clinical findings

On examination, a 2.5 cm tender swelling with ill-defined borders was noted over the left parotid gland. Examination of the temporomandibular joint was normal.

### Diagnostic assessment

**Fig. 1:**
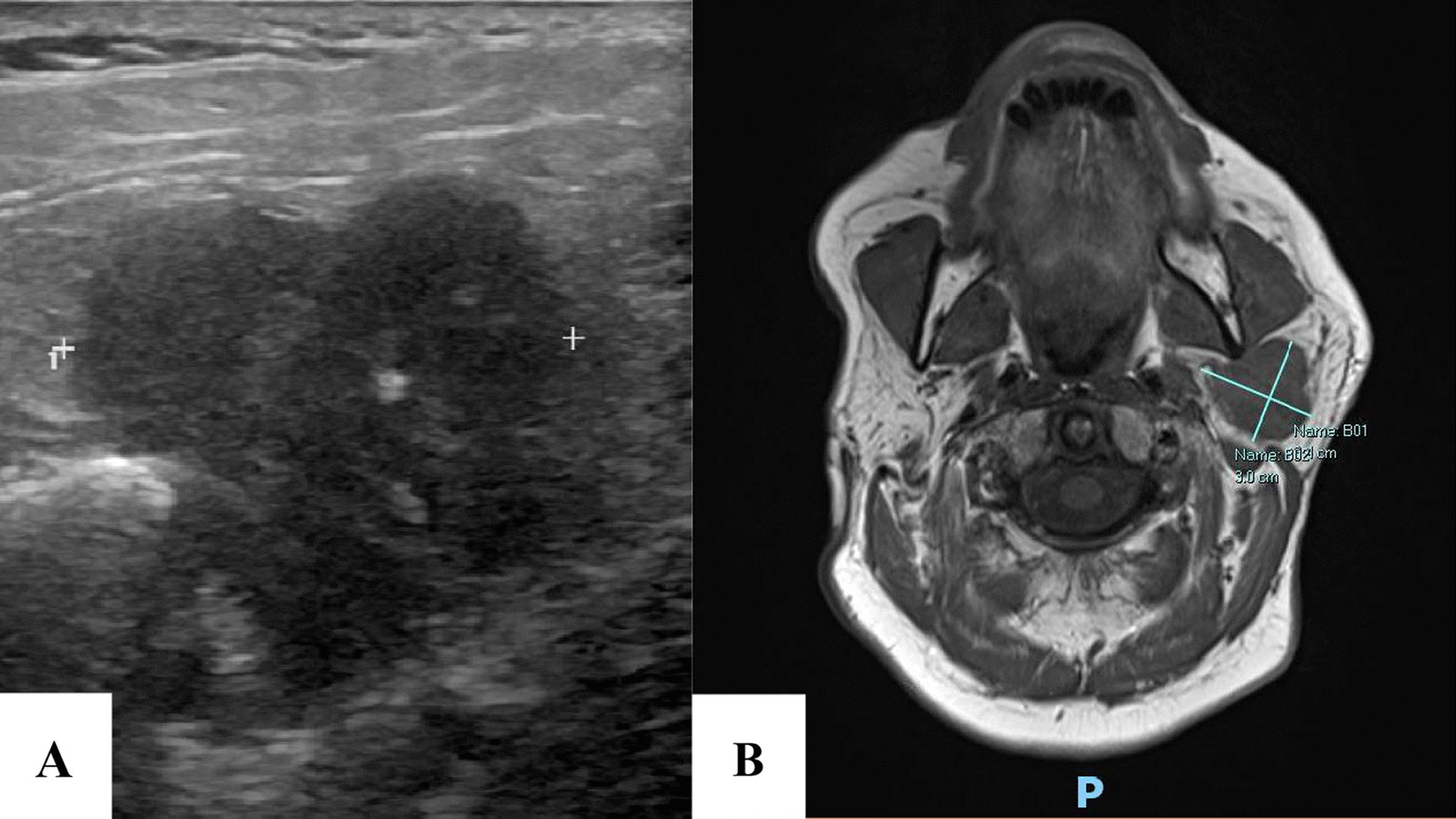
Ultrasonography (**A**) showed a hypoechoic mass in the left parotid, 2.8 cm in greatest dimension with poorly defined borders. Magnetic Resonance Imaging (**B**) showed a well-defined cystic and/or necrotic mass involving the left parotid gland, 3.4 × 3.0 × 3.0 cm

Ultrasonography showed a hypoechoic mass in the left parotid, 2.8 cm in the greatest dimension with poorly defined borders (Fig. 1A). A magnetic resonance imaging was done which showed a well-defined cystic and/or necrotic mass involving the left parotid gland, 3.4 × 3.0 × 3.0 cm (Fig. 1B). The lesion involved the superficial lobe of the parotid gland and passed through the stylomandibular tunnel to involve the lateral aspect of the deep lobe of the gland and demonstrated heterogenous increased and decreased STIR signal, with heterogenous enhancement with areas of cystic change/necrosis.

Ultrasound-guided fine needle aspiration was done using 21- and 25-gauge needles and reported as atypia of undetermined significance-Milan classification 3. The smears showed scattered clusters and single cells with increased nuclear to cytoplasmic ratio and some hyperchromasia in a background of debris with lymphocytes and neutrophils, most likely representing necrosis. While an inflammatory or cystic process is possible, the possibility of either primary or metastatic malignancy could not be excluded.

### Therapeutic intervention

**Fig. 2:**
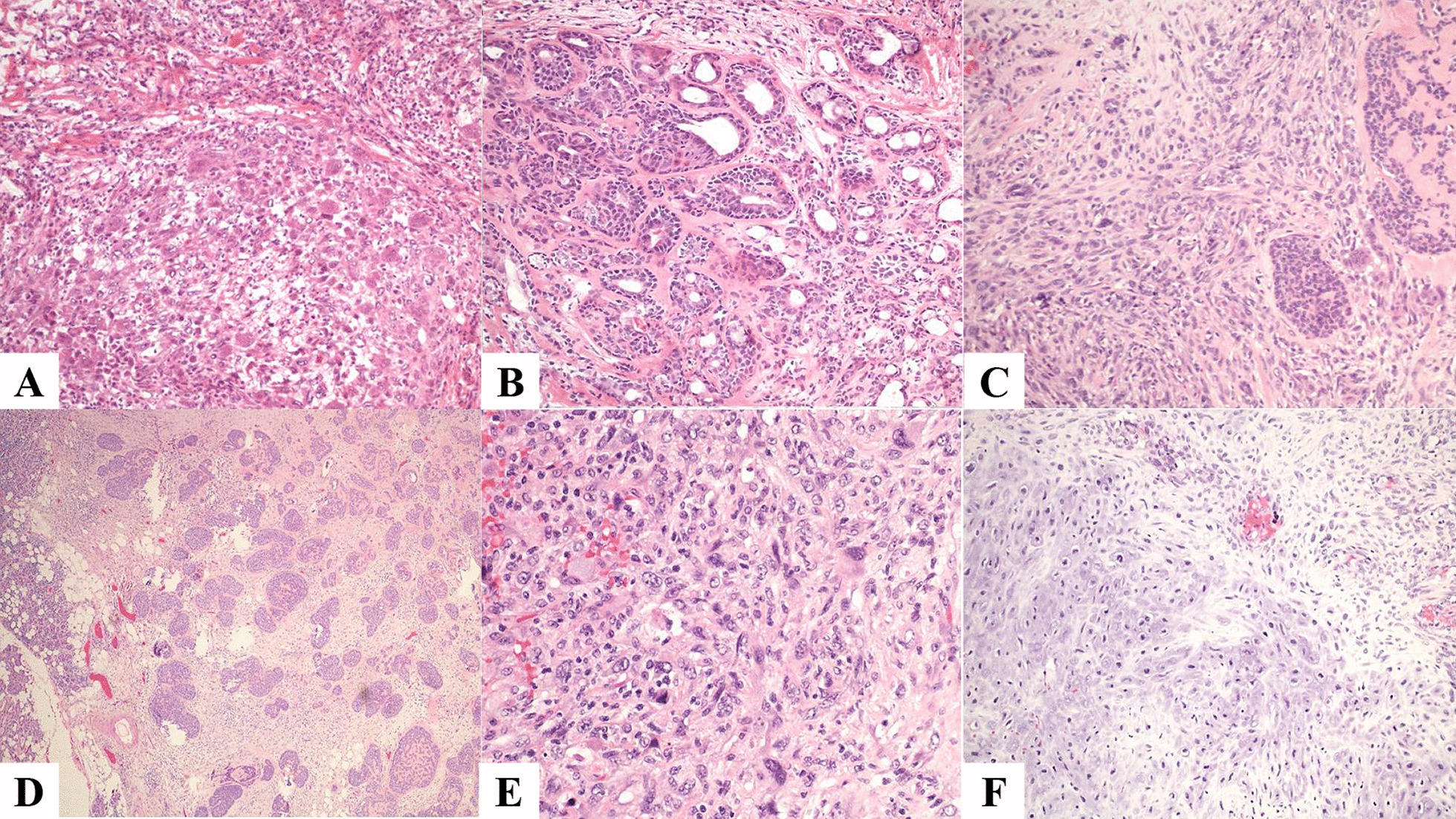
Hematoxylin and eosin-stained photomicrographs: Frozen section (**A**, **B**, 20X) showed an adenocarcinoma with poorly differentiated component. Permanent sections showed an adenoid cystic carcinoma with adjacent high-grade sarcoma (**C**, 20X). The adenoid cystic showed predominant tubular and cribriform areas (**D**, 20X). Sarcomatous areas showed marked atypia with scattered mitoses (**E**, 20X) with focal chondroid areas (**F**, 20X)

This was followed by a left total parotidectomy, left selective neck dissections levels 2 and 3, and soft tissue reconstruction using sternocleidomastoid muscle. An intraoperative consultation was done and was reported as adenocarcinoma with a poorly differentiated component (Fig. 2A, B). The deep lobe showed a 3.5 × 2.9 × 2.9 cm white tan, focally calcified lesion abutting the resection margin. On microscopy, the lesion was composed of distinct carcinomatous and sarcomatous elements (Fig. 2C) both involving the adjacent soft tissue and adipose tissue. The carcinomatous component was composed of adenoid cystic carcinoma with predominant tubular and cribriform growth patterns, and focal solid growth patterns (Fig. 2D). Also, there was a high-grade spindle cell neoplasm with occasional giant cells and scattered mitoses, and extensive necrosis. Occasional foci of chondroid differentiation were noted (Fig. 2E, F).

**Fig. 3:**
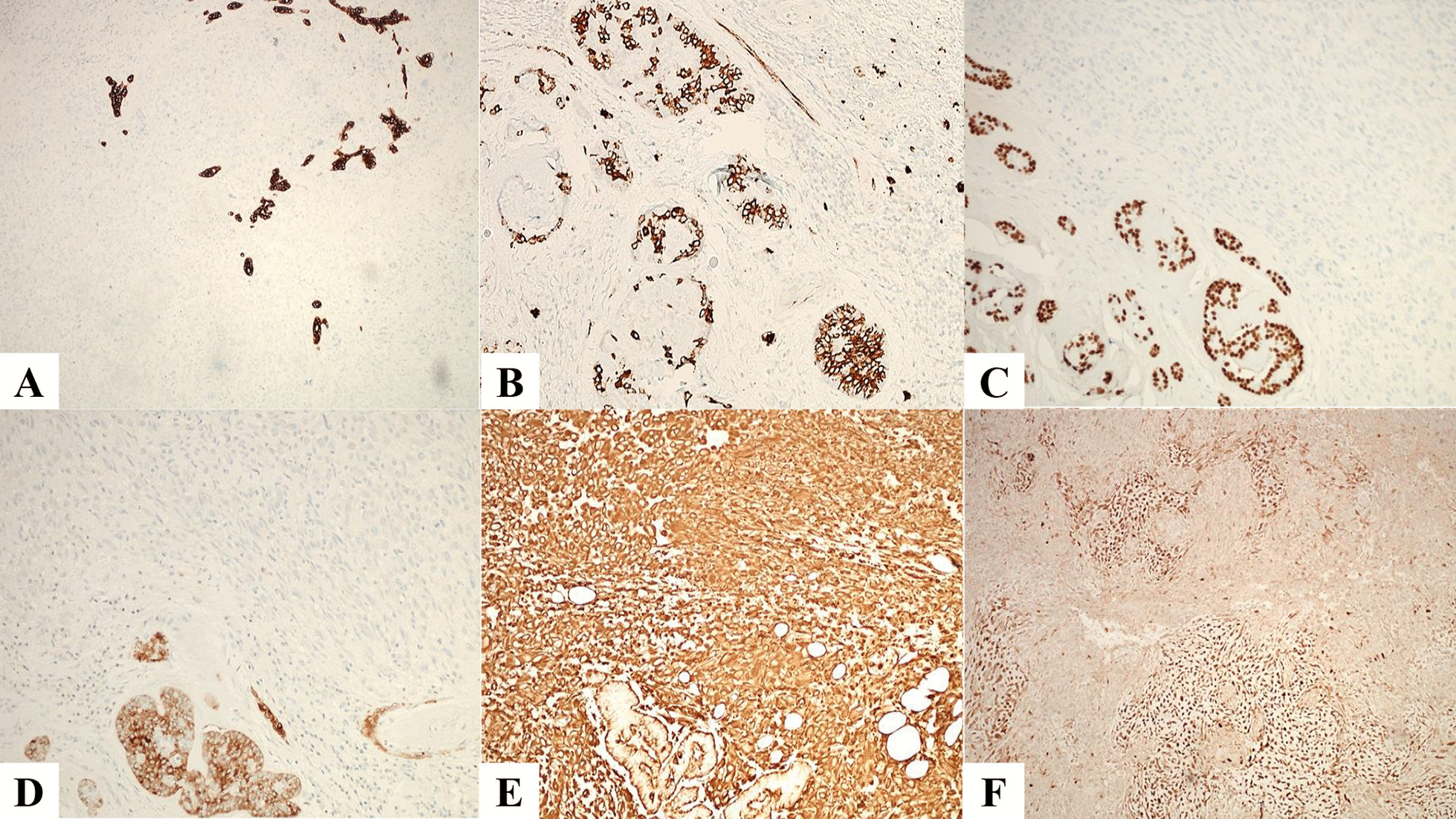
Immunohistochemical stains performed: CAM5.2 (**A**, 20X), CD117 (**B**, 20X), p63 (**C**, 20X), SMMH (**D**, 20X) were positive in adenoid cystic carcinoma, but were negative in sarcoma component. Vimentin (**E**, 20X) was strongly diffuse positive in the sarcoma component. S-100 (**F**, 20X) highlighted the cartilaginous component

Immunohistochemical stains performed on the tumor showed CAM 5.2, CD117, p63, SMMH (Fig. 3A–D), p40, CK7, pancytokeratin AE1/AE3, and GATA-3 positive in adenoid cystic carcinoma, but negative in sarcoma component. Androgen receptors, CD57, and calponin were negative. The sarcoma component was positive for vimentin (Fig. 3E) and S-100 highlighted the foci of chondroid differentiation (Fig. 3F). Ki-67 proliferation index was 50–70% in the sarcomatous area. No foci of pleomorphic adenoma were identified. It was suggested that this morphology could represent a carcinosarcoma ex-pleomorphic adenoma or de novo carcinosarcoma. Lymph nodes were negative for metastatic carcinoma.

### Follow-up and outcomes

The patient is currently undergoing radiotherapy of the tumor bed and skull base due to the propensity of perineural invasion of the adenoid cystic component.

## Discussion

Carcinosarcoma of the parotid gland is extremely rare and is first reported in 1951 by Kirklin *et al.* [4]. The term malignant mixed tumor was coined in 1976 by King Jr [5] and less than 100 cases reported to date in literature [6]. Carcinosarcomas can arise from a pre-existing pleomorphic adenoma (PA) or may occur de novo [7]. These are high-grade tumors with an aggressive clinical course with distant metastases being reported in 54% of cases [8].

Gupta *et al.* reviewed 66 cases of carcinosarcomas in the salivary glands over 42 years (1973–2015) and found that these tumors most commonly occur in the sixth to seventh decade of life with a male preponderance. The parotid was the most common location (78%) and the mean size of the tumor at presentation was more than 4 cm with extra-parenchymal extension in 43.9% of cases [9].

There are two theories postulating the origin of these tumors. The convergence hypothesis suggests these tumors to be arising from two or more polyclonal stem cells while the divergent theory proposes they arise from a totipotent stem cell and differentiate into distinct epithelial and mesenchymal elements [10]. The genomic profile of the carcinomatous and sarcomatous elements was studied by Vekony *et al.* using oligonucleotide microarray-based comparative genomic hybridization. 75% homology was seen between the two components suggesting the divergent hypothesis that these components are clonally related [11].

The most common sarcomatous element is chondrosarcoma as was seen in our case, followed by fibrosarcoma, leiomyosarcoma, osteosarcoma, and liposarcoma [12]. The most common carcinomatous elements seen are adenocarcinoma, undifferentiated carcinoma, and squamous cell carcinoma [3]. Myoepithelial carcinoma and epithelial–myoepithelial carcinoma have also been reported [13]. The adenoid cystic carcinoma seen in our case was predominant tubular and cribriform growth patterns and focal solid growth patterns. On histomorphology, a diagnosis of carcinoma ex-pleomorphic adenoma, a sarcomatoid variant of salivary duct carcinoma, and myoepithelial carcinoma were considered. Immunohistochemistry is helpful in the diagnosis of such lesions. The adenoid cystic carcinoma was stained for both ductal (CK7, CAM 5.2) and myoepithelial (p63, p40, cytokeratin, SMMH) markers and CD117. The sarcomatous component was negative for all epithelial markers, CD57, AR, and calponin. It was positive for vimentin and showed focal S-100 positivity in the chondroid component. This immunohistochemical profile in the absence of any history of pre-existing parotid lesion and absence of histological evidence of pleomorphic adenoma was suggestive of primary carcinosarcoma of the parotid gland.

Fine needle aspiration cytology has not been extensively studied in these tumors. Previously cases have been reported as suspicious and atypical on cytology and a final diagnosis was given based on resection specimens [3, 14]. In our case, cellular atypia and hyperchromasia in a background of necrosis prompted the lesion to be classified as Milan 3.

Fowler *et al.* studied tumor suppressor genes (3p, 5q, 9p, 17p, and 18q) for loss of heterozygosity in malignant mixed tumors of the salivary gland. It was noted that loss of heterozygosity of 17q21 and 9p21 was seen only in carcinosarcomas de novo as compared to carcinoma ex pleomorphic adenoma. The sarcomatous areas had a higher mean fractional allelic loss as compared to the carcinomatous areas suggesting additional mutations in those areas [13]. Katsakhyan *et al.* reported a case of carcinosarcoma harboring PLAG1 translocation with an adjacent PA with an HMGA2 translocation [15]. As these translocations are mutually exclusive, it was concluded that the carcinosarcoma most likely came from another PA with PLAG1 mutation or originated de novo, thereby suggesting that the presence of PA is not conclusive of carcinosarcoma ex pleomorphic adenoma.

The imaging modality of choice is Magnetic Resonance Imaging as it helps determine the involvement of the deep lobe, facial nerve, and surrounding soft tissues [16]. These tumors have a poor prognosis with overall 2-year and 5-year survival having been reported as 68.1% and 37.2% respectively. The median survival reported by Gupta *et al.* was 38 months while some studies have reported medical survival as low as 10 months. Distant metastases have been reported in up to 54% of cases and are an independent predictor of poor survival [8, 9]. The treatment of choice is radical parotidectomy followed by postoperative radiotherapy. Staffieri *et al.* demonstrated a significant decrease in recurrence rate following surgery followed by radiotherapy as compared to surgery alone. The most common site for metastases is the lungs followed by hilar and cervical lymph nodes and is mostly hematogenous [9].

## Conclusion

Carcinosarcoma is a high-grade aggressive lesion with a poor prognosis and should be treated aggressively. More studies are needed to understand the origin of these tumors. We reported a case of carcinosarcoma with carcinomatous component being adenoid cystic carcinoma and is the first case of an adenoid cystic carcinoma with chondrosarcoma to the best of our knowledge.

## Data Availability

Supporting data is available for future validation.
